# Angiotensin Receptor Blockers Decrease the Risk of Major Adverse Cardiovascular Events in Patients with End-Stage Renal Disease on Maintenance Dialysis: A Nationwide Matched-Cohort Study

**DOI:** 10.1371/journal.pone.0140633

**Published:** 2015-10-21

**Authors:** Chung-Wei Yang, Nian-Sheng Tzeng, Yun-Ju Yin, Chien-Hsun Li, Hung-An Chen, Shih-Hsiang Chiu, Shinn-Ying Ho, Hui-Ling Huang

**Affiliations:** 1 Division of Nephrology, Department of Medicine, National Taiwan University Hospital Hsin-Chu Branch, Hsin-chu, Taiwan; 2 Department of Psychiatry, Tri-Service General Hospital, School of Medicine, National Defense Medical Center, Taipei, Taiwan; 3 Student Counseling Center, National Defense Medical Center, Taipei, Taiwan; 4 Institute of Bioinformatics and Systems Biology, National Chiao Tung University, Hsinchu, Taiwan; 5 Division of Neurosurgery, Department of Surgery, National Taiwan University Hospital Hsin-Chu Branch, Hsinchu, Taiwan; 6 Department of Biological Science and Technology, National Chiao Tung University, Hsinchu, Taiwan; National Health Research Institutes, TAIWAN

## Abstract

**Background:**

Major adverse cardiovascular events (MACE) cause the leading cause of morbidity and mortality in patients with end-stage renal disease (ESRD) on maintenance Hemodialysis (HD) or peritoneal dialysis (PD). Many randomized-controlled trials (RCTs) have proved that angiotensin receptor blockers (ARBs) can reduce the risk of MACE in the people with normal or impaired kidney function without dialysis. This study seeks to clarify whether ARBs therapy could also attenuate this risk in patients with ESRD on maintenance dialysis.

**Materials and Methods:**

The National Health Research Institute provided a database of one million random subjects for the study. A random sample was taken of 1800 patients ≥18 years y/o with ESRD on dialysis without a history of MACE and use of ARBs within 6-months prior to enrollment. Cox proportional hazard regression analysis was used to identify the risk factors and compute the hazard ratios accompanying 95% confidence intervals.

**Results:**

In these 1800 patients, 1061 had never used ARBs, while 224 had used them for 1–90 days, and 515 had used them for more than 90 days. We found that ARBs significantly decrease the incidences of acute myocardial infarctions (AMI), coronary artery diseases (CAD) requiring coronary stent or percutaneous transluminal coronary angioplasty (PTCA), peripheral artery disease (PAD) requiring percutaneous transluminal angioplasty (PTA), and acute stroke. Cumulative prescription days of ARBs beyond 365–760 days or more were found to be negatively correlated with incidence of MACEs. For patients with dual comorbidity (i.e., mellitus and hyperlipidemia), 91–365 cumulative prescription days might also attenuate the risk.

**Conclusions:**

For patients on maintenance dialysis, the use of ARBs could significantly attenuate the risk of major cardiovascular events: AMI, acute stroke, and PAD requiring PTA.

## Introduction

At 2,902 per million population, Taiwan’s end-stage renal disease (ESRD) rate is the highest in the world [1]. Furthermore, patients with ESRD have a much higher risk of cardiovascular disease [2, 3], and are 10–30 times more likely to die of cardiovascular disease than non-dialysis patients of the same age [4, 5]. In total, 44% of ESRD patients die from cardiovascular disease [6]. A study in the US found that 63% of patients with chronic kidney disease (CKD) suffer from cardiovascular disease, as opposed to only 5.8% in non-CKD patients [7]. In addition, the incidence of certain cardiovascular diseases increases with CKD severity. For example, the incidence rate of myocardial infarction in stages I and II is 5.3%, but 10.1% in stages III and IV [5]. This high morbidity and mortality of cardiovascular diseases has emerged as a significant challenge in treating ESRD patients.

Hypertension is an important risk factor for cardiovascular disease, and is estimated to afflict up to 80% of the CKD population [8, 9]. Angiotensin receptor blockers (ARBs), an effective and well-tolerated orally-active antihypertensive drug, act mainly by blocking the angiotensin II receptor, type 1 (AT_1_), thereby relaxing the vascular smooth muscle, increasing salt excretion, decreasing cellular hypertrophy and inducing an antihypertensive effect without modifying the heart rate or cardiac output [10, 11]. Reports show ARBs have benefits for patients with normal kidney function or CKD on protecting target organs, such as heart [12, 13] or kidney [14–16]. Among the patients on maintenance dialysis, we do not have adequate evidences to prove whether the ARBs have the same effects, especially on cardiovascular system. We therefore conducted a nationwide, population-based study to clarify whether ARB therapy in chronic dialysis patients could attenuate the risk of major adverse cardiovascular events (MACEs).

The Taiwan National Health Insurance Research Database (NHIRD) is used to clarify whether ARBs could attenuate the risk of MACE in chronic dialysis patients in a five year follow-up (2000–2005). As of June 2009, Taiwan’s National Health Insurance (NHI) Program covers more than 99% of the population, and 97% of medical providers [17]. Previous studies have found the NHIRD to be a valid resource for medical research [18, 19].

## Materials and Methods

### Database

In Taiwan, the patients who need chronic dialysis are made by nephrologists and NHI administration will review then issue a catastrophic illness card to each patient receiving hemodialysis (HD) or peritoneal dialysis (PD).

This population-based cohort study used healthcare data from the Longitudinal Health Insurance Database 2005 (LHID2005), which was randomly sampled from the National Health Insurance Research Database (NHIRD). The NHIRD has prospectively collected data since the implementation of Taiwan’s National Health Insurance (NHI) in 1995. It covers both outpatient and inpatient services for approximately 99% of entire 23 million population of Taiwan. The LHID2005 contains 1,000,000 original claims data which were randomly sampled from the year 2005 registry of all beneficiaries under the NHI program. It comprises comprehensive health care data including encrypted patient identification number, demographic data, outpatient/inpatient visits, diagnosis codes and details of prescriptions. Diseases were coded using the ICD-9-CM (International Classification of Diseases, 9th Revision, Clinical Modification) diagnosis codes. Dialysis patients were identified based on diagnosis codes and the Catastrophic Illness Registry. The Catastrophic Illness Registry contains records of patients, who possess catastrophic illness cards issued by NHI administration. The records for chronic dialysis patients receiving either hemodialysis (HD) or peritoneal dialysis (PD) cards are updated every year to ensure the dialysis status. Personal medical information in the database had been encrypted for privacy. There were no statistically significant differences in the distribution of sex, age, number of births per year or health-care costs between patients in the database.

### Population

From July 1, 2000 to December 31, 2005 in LHID2005, a total of 2722 ESRD patients were given catastrophic illness cards. As for ESRD diagnosis, we used both diagnosis codes (ICD-9-CM code: 585) and the Catastrophic Illness Registry. Patients were excluded from consideration if they 1) were aged < 18 years, 2) had ARB records within the previous six months as wash-out periods, and 3) had MACE prior to enrollment. The final sample included 1800 chronic dialysis patients.

**Fig 1:**
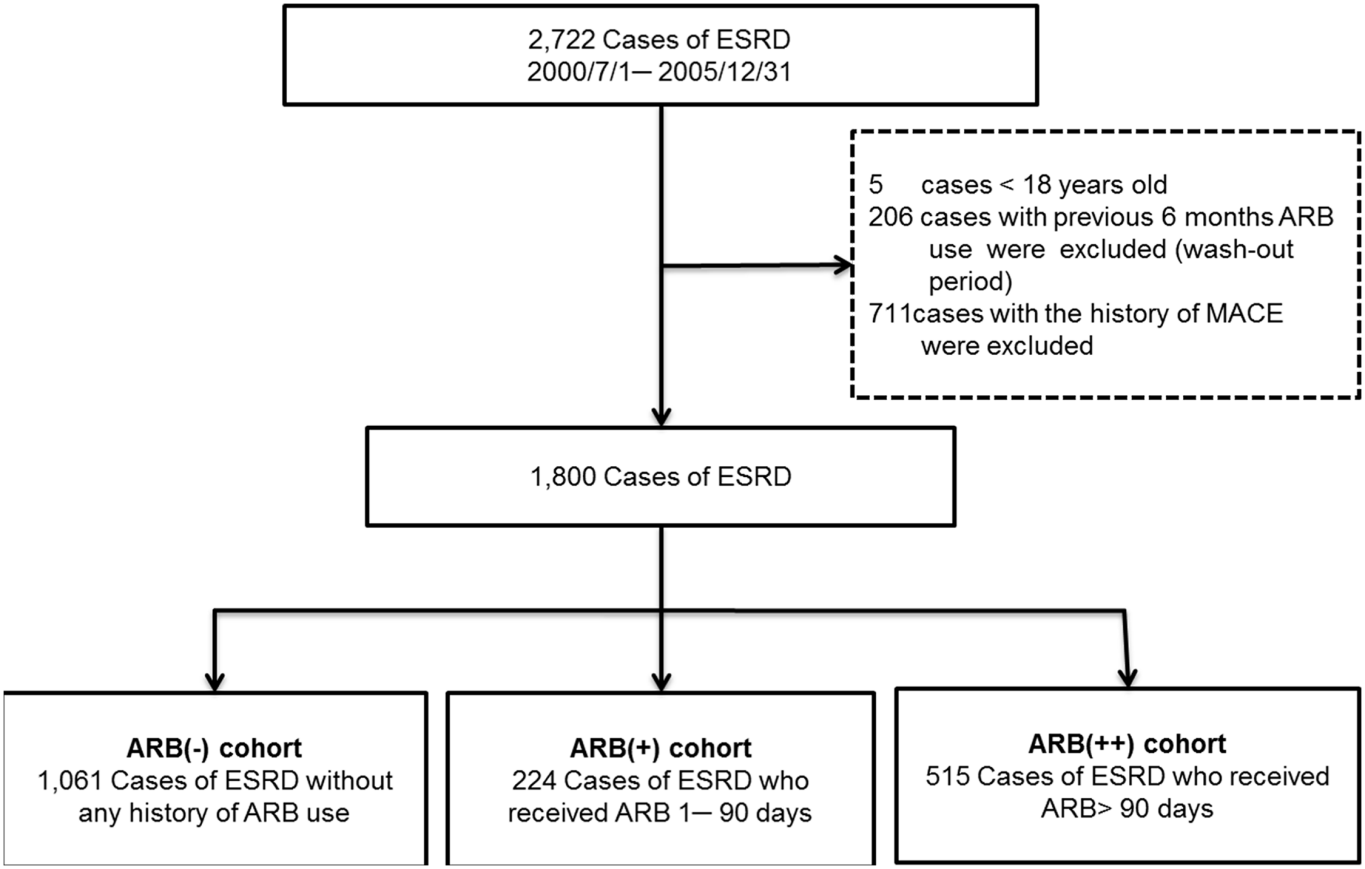
Flowchart of selecting study subjects.

Patients were divided into ARB user and non-user groups according to ARB prescriptions between follow-ups. For patients who never used ARBs, the index date of the follow-up period was set as the date of beginning dialysis, while that for patients who used ARBs regularly was set as the first ARB prescription date. In ARB user group, we define the treatment of ARBs with 90 or fewer cumulative prescription days as short-term use group (ARB (+)), and those more than 90 cumulative prescription days as long-term use group (ARB (++)). Fig 1 illustrates the population selection process.

The study endpoint was the occurrence of first MACE, including 1) acute myocardial infarction (AMI, ICD-9-CM codes 410.xx), 2) acute stroke (ICD-9-CM codes 430.xx-436.xx), 3) peripheral arterial disease (PAD, ICD-9-CM codes 440.2x, 440.3x, 444.2x, 444.8x,) combined with percutaneous transluminal angioplasty (PTA, treatment codes 33074A, 33074B, 33115B), 4) coronary artery disease (CAD) combined with stent implantation and/or percutaneous transluminal coronary angioplasty (PTCA, treatment codes 33076A, 33076B, 33077A, 33077B, 33078A, 33078B). Every subject was followed up until the study endpoint or 5 years after enrollment.

NHIRD information was collected for comorbidities, including hypertension (ICD-9-CM codes 401.xx–405.xx), diabetes mellitus (ICD-9-CM codes 250.xx), hyperlipidemia (ICD-9-CM codes 272.0x-272.4x), and Chronic obstructive pulmonary disease (COPD, ICD-9-CM codes 491.xx-492.xx, 494.xx, and 496.xx). Age was entered as a categorical variable (18–39, 40–49, 50–59, 60–69, and 70 years or older).

### Statistical analysis

Differences in distribution of age, gender, and comorbidities between with ARB group and non-ARB group were compared using the chi square test for categorical variables. Kaplan-Meier analysis was used to assess cumulative hazard, with significance based on the log-rank test. Multiple regression analysis was conducted using Cox proportional hazard regression analysis to identify risk factors and compute the hazard ratios (HRs) accompanying 95% confidence intervals (95% CIs). A two-sided P < 0.05 was considered statistically significant. All statistical analyses were performed using SPSS software (Version 19.0, SPSS, Inc., Chicago, IL, USA) and Microsoft SQL Server 2008 (Redmond, Seattle, WA, USA) for data management.

### Ethical statement

This study conforms to Declaration of Helsinki protocols, and was approved from a full review by the Institutional Review Board of Cathay General Hospital (permission code: CGH-OP102002). All the data set consists of de-identified secondary data, and was released without restrictions for research purposes.

## Results

Table 1 shows demographic distributions for the 1800 patients enrolled in this study. Among them, 1061 were in the ARB(-) group (i.e., not treated with ARB at all), 224 were in the ARB(+) group (short-term use group), and 515 were in the ARB(++) group (long-term use group). The sex ratio of ARB(-) group (female 52.6% and male 47.4%) was similar to those in previous studies which showed that female patients had a higher incidence [20, 21]. No significant gender difference was found between two groups. However, several comorbidities were more prevalent among the ARB(++) and ARB(+) groups, including hypertension (95.50%, 92.40%, and 50.60%; P<0.001), diabetes (52.80%, 55.40%, and 23.60%; P<0.001), hyperlipidemia (39.20%, 29.90%, and 17.30%; P<0.001), and COPD (21.90%, 24.10%, and 13.10%; P<0.001). ARB users had a higher prevalence of these cardiovascular comorbidities than non-users. The ARB types included losartan, valsartan, irbesartan, candesartan, olmesartan, telmisartan and eprosartan.

**Table 1:** Demographics and comorbidities among ARB(-), ARB(+), and ARB(++) groups.

Variables	ARB(-)		ARB(+)		ARB(++)		P-value
	*N*	%	*N*	%	*N*	%	
Gender							0.096
female	558	52.60%	100	44.60%	264	51.30%	
male	503	47.40%	124	55.40%	251	48.70%	
Age group							<0.05
18–39	139	13.10%	23	10.30%	69	13.40%	
40–49	234	22.10%	36	16.10%	90	17.50%	
50–59	220	20.70%	53	23.70%	140	27.20%	
60–69	238	22.40%	54	24.10%	129	25.00%	
≥70	230	21.70%	58	25.90%	87	16.90%	
Comorbidity							
Hypertension	537	50.60%	207	92.40%	492	95.50%	<0.001
Diabetes	250	23.60%	124	55.40%	272	52.80%	<0.001
Hyperlipidemia	184	17.30%	67	29.90%	202	39.20%	<0.001
COPD	139	13.10%	54	24.10%	113	21.90%	<0.001
Hyperkalemia	64	6.00%	34	15.20%	70	13.60%	<0.001
Type of ARBs							
Losartan	-	-	94	41.96%	288	55.92%	
Valsartan	-	-	98	43.75%	305	59.22%	
Irbesartan	-	-	43	19.20%	165	32.04%	
Candesartan	-	-	7	3.13%	28	5.44%	
Olmesartan	-	-	2	0.89%	19	3.69%	
Telmisartan	-	-	7	3.13%	49	9.51%	

COPD = Chronic obstructive pulmonary disease

**Fig 2:**
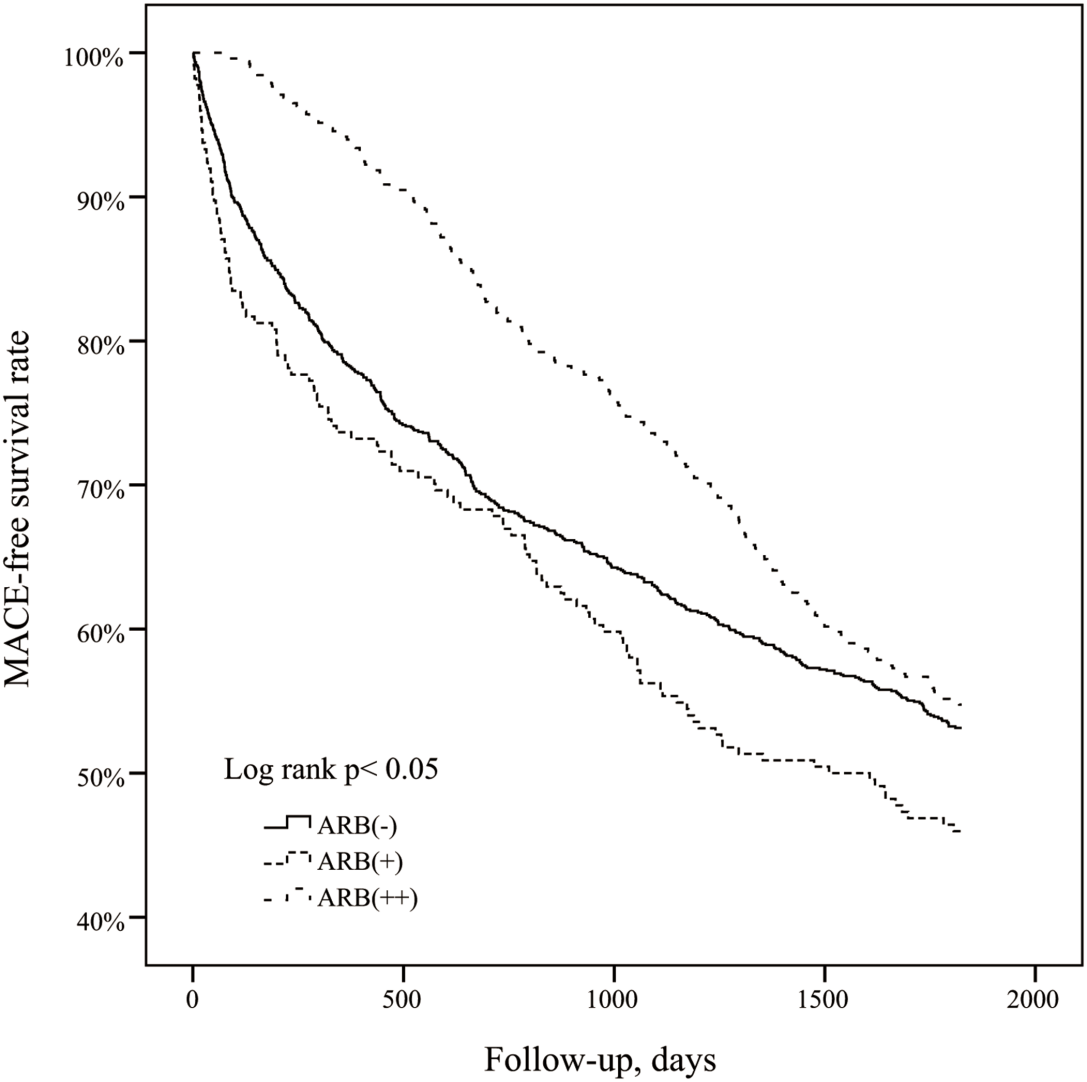
The Kaplan-Meier curve of the MACE-free rate.

During the 5 years follow-up, 851 of the subjects developed incident MACE, including 233 from the ARB(++) group, 121 from the ARB(+) group, and 497 from the ARB(-) group. The Kaplan–Meier curve plot presented in Fig 2 shows the protective effect of long-term ARB use (ARB (++) group) against the MACE occurrence. (Log-rank test P-value < 0.05). Table 2 also shows the ARB (++) group has significantly lower the new incidence of MACE (adjusted HR = 0.68, 95% CI = 0.57–0.82, P < 0.001). However, we cannot find the benefits in the ARB (+) group compared with ARB (-) group in Fig 2 and Table 2.

**Table 2:** Crude and adjusted HR for the development of new-onset MACE.

Variables	Crude HR (95% CI)^a^ ^,^ ^c^	P-value	Adjusted HR (95% CI)^a^ ^,^ ^b^ ^,^ ^c^	P-value
ARB treatment		<0.05		<0.001
ARB(-)	1		1	
ARB(+)	1.24(1.02–1.51)*		0.99(0.80–1.22)	
ARB(++)	0.85(0.73–1.00)*		0.68(0.57–0.82)***	
Gender		0.645		0.581
Female	1			
Male	1.03(0.90–1.18)		1.04(0.91–1.19)	
Age group		<0.001		<0.001
18–39	1		1	
40–49	1.29(0.96–1.74)		1.18(0.87–1.59)	
50–59	1.84(1.39–2.44)***		1.63(1.23–2.17)*	
60–69	2.58(1.96–3.39)***		2.28(1.73–3.02)***	
≥70	2.81(2.13–3.70)***		2.45(1.85–3.25)***	
Comorbidity				
Hypertension	1.20(1.03–1.40)	<0.05	1.12(0.94–1.33)	0.196
Diabetes	1.62(1.41–1.85)	<0.001	1.40(1.21–1.63)	<0.001
Hyperlipidemia	1.27(1.09–1.47)	<0.05	1.27(1.09–1.49)	<0.05
COPD	1.24(1.05–1.47)	<0.05	1.01(0.85–1.21)	0.888

^a^ HR = hazard ratio; CI = confidence interval.

^b^ Adjusted for gender, age group, and comorbidities.

^c^ *P < 0.05; ***P < 0.001.

In addition, we also find that age > 50 years (aged 50–59: adjusted HR = 1.63, 95% CI = 1.23–2.17, P<0.05), 60–69: adjusted HR = 2.28, 95% CI = 1.73–3.02, P<0.001, and ≥70: adjusted HR = 2.45, 95% CI = 1.85–3.25, P<0.001), diabetes mellitus (adjusted HR = 1.40, 95% CI = 1.21–1.63, P < 0.001) and hyperlipidemia (adjusted HR = 1.27, 95% CI = 1.09–1.49, P < 0.05) were significantly associated with higher risk of MACE in chronic dialysis patients. Hypertension (adjusted HR = 1.12, 95% CI = 0.94–1.33, P = 0.196) and COPD (adjusted HR = 1.01, 95% CI = 0.85–1.21, P = 0.888) were not the risk factors for MACE in this patient group.

Table 3 shows the association between the effects of decreasing risk of MACE and the cumulative prescription days of ARBs. The number of cumulative prescription days was divided as 1–90, 90–365, 366–730, 731–7095, 1096–1460, and 1460–1825 days. Compared to non-users, no significant risk decrease was found for ARB users with 1–90 and 91–365 cumulative prescription days (1–90 days, HR = 0.99, 95% CI = 0.81–1.23; 91–365 days, HR = 0.87, 95% CI = 0.71–1.08). However, for ARB users additional cumulative prescription days, a significantly decreased risk of MACE was found in contrast to non-users as follows: 366–730 days (HR = 0.72, 95% CI = 0.55–0.95, P < 0.05), 731–1095 days (HR = 0.51, 95% CI = 0.35–0.75, P < 0.001), 1096–1460 days (HR = 0.34, 95% CI = 0.18–0.64, P < 0.05) and 1460–1825 days (HR = 0.06, 95% CI = 0.01–0.45, P < 0.05). Thus, cumulative prescription days over 365 days will significantly reduce the risk of MACE in chronic dialysis patients. The greater the cumulative number of prescription days, the lower the risk of MACE.

**Table 3:** Risk of MACE for patients with different cumulative prescription days of ARB.

Variables	N	Event, n	Adjusted HR (95% CI)^a^ ^,^ ^b^ ^,^ ^c^	P-value
Duration				<0.001
non-users	1061	497	1	
1–90 days	224	121	1.00(0.81–1.23)	
91–365 days	237	128	0.87(0.71–1.08)	
366–730 days	136	64	0.72(0.55–0.95)*	
731–1095 days	78	30	0.51(0.35–0.75)***	
1096–1460 days	42	10	0.34(0.18–0.64)*	
1460–1825 days	22	1	0.06(0.01–0.45)*	

^a^ HR = hazard ratio; CI = confidence interval.

^b^ Adjusted for gender, age group, and comorbidities.

^c^ *P < 0.05; ***P < 0.001.

Table 4 compares the ARB-associated decrease risk of MACE for patients with different comorbidity loadings. For these patients with hypertension only, durations of ARB use more than 1095 cumulative prescription days were associated significantly decreased risk of MACE (HR = 0.26, 95% CI = 0.08–0.81, P < 0.05); for patients with both hypertension and diabetes mellitus, while 366–1095 cumulative prescription days (HR = 0.67, 95% CI = 0.45–1.00, P < 0.05) and more than 1095 days (HR = 0.08, 95% CI = 0.01–0.59, P < 0.05) were associated with significantly decreased risk of MACE. For patients with hypertension and hyperlipidemia, 366–1095 cumulative prescription days (HR = 0.33, 95% CI = 0.14–0.82, P < 0.05) was significantly associated with reduced risk of MACE, but no more benefits with cumulative prescription days > 1095 days (HR = 0.62, 95% CI = 0.18–2.18). For patients with hypertension, diabetes and hyperlipidemia, 91–365 days (HR = 0.59, 95% CI = 0.39–0.89, P < 0.05), 366–1095 days (HR = 0.49, 95% CI = 0.31–0.77, P < 0.05), and >1095 days (HR = 0.22, 95% CI = 0.08–0.62, P < 0.05) was significantly associated with reduced risk of MACE.

**Table 4:** Comorbidity-relation analysis for MACE.

Comorbidity	N	Event, n	Adjusted HR (95% CI)^a^ ^,^ ^b^ ^,^ ^c^	P-value
No comorbidity				0.811
non-users	413	178	1	
1–90 days	12	4	0.64(0.24–1.72)	
91–365 days	8	2	0.53(0.13–2.14)	
366–1095 days	6	2	0.89(0.22–3.66)	
>1095 days	1	0	-	
Hypertension only				<0.05
non-users	291	121	1	
1–90 days	73	36	1.33(0.92–1.93)	
91–365 days	74	31	0.88(0.59–1.31)	
366–1095 days	55	17	0.69(0.42–1.15)	
>1095 days	26	3	0.26(0.08–0.81)*	
Hypertension+ Diabetes				<0.05
non-users	130	78	1	
1–90 days	68	36	0.84(0.57–1.26)	
91–365 days	54	35	1.07(0.71–1.61)	
366–1095 days	69	36	0.67(0.45–1.00)*	
>1095 days	13	1	0.08(0.01–0.59)*	
Hypertension+ Hyperlipidemia				<0.05
non-users	54	25	1	
1–90 days	14	9	2.04(0.86–4.84)	
91–365 days	32	14	0.95(0.49–1.86)	
366–1095 days	30	6	0.33(0.14–0.82)*	
>1095 days	10	3	0.62(0.18–2.16)	
Hypertension+ Diabetes+ Hyperlipidemia				<0.05
non-users	62	50	1	
1–90 days	52	35	0.75(0.48–1.16)	
91–365 days	67	45	0.59(0.39–0.89)*	
366–1095 days	51	32	0.49(0.31–0.77)*	
>1095 days	11	4	0.22(0.08–0.62)*	

^a^ HR = hazard ratio; CI = confidence interval.

^b^ Adjusted for gender and age group.

^c^ *P < 0.05; ***P < 0.001.

In Table 5, we discuss the relationship between the different types of MACE and the ARB (++) group. Long-term use of ARBs was associated with lower risk of developing acute myocardial infarctions (HR = 0.58, 95% CI = 0.38–0.88, P < 0.05), acute stroke (HR = 0.67, 95% CI = 0.50–0.90, P < 0.05) and peripheral artery disease requiring percutaneous transluminal angioplasty (HR = 0.49, 95% CI = 0.25–0.95, P < 0.05), but not for patients with coronary artery disease requiring coronary stent and/or percutaneous transluminal coronary angioplasty (PTCA).

**Table 5:** The development of different types of MACE.

ARB group	N	Event, n	Adjusted HR (95% CI)^a^ ^,^ ^b^ ^,^ ^c^
AMI			
ARB(-)	643	79	1
ARB(+)	124	21	0.82(0.50–1.37)
ARB(++)	320	38	0.58(0.38–0.88)*
Acute stroke			
ARB(-)	759	195	1
ARB(+)	145	42	0.90(0.63–1.29)
ARB(++)	365	83	0.67(0.50–0.90)*
PAD			
ARB(-)	715	151	1
ARB(+)	139	36	0.99(0.67–1.47)
ARB(++)	363	81	0.81(0.60–1.11)
PTA			
ARB(-)	613	49	1
ARB(+)	108	5	0.46(0.18–1.21)
ARB(++)	295	13	0.49(0.25–0.95)*
PTCA			
ARB(-)	574	10	1
ARB(+)	109	6	1.33(0.44–4.01)
ARB(++)	294	12	0.94(0.37–2.42)

^a^ HR = hazard ratio; CI = confidence interval.

^b^ Adjusted for gender, age, and comorbidities.

^c^ *P < 0.05; ***P < 0.001.

## Discussion

Our aim focuses on the possible effects of MACE protection by the use of ARBs in chronic dialysis patients. The use of angiotensin-converting-enzyme inhibitors (ACEIs) may interfere the results, because both ACEIs and ARBs are the blockades of the renin-angiotensin-aldosterone system. But the use of ACEIs in dialysis patients is not very common due to their disturbing side effects: cough and hyperkalemia. On the other hand, because of lots of generic drugs and brand drugs in Taiwan’s medical market, we cannot make a good analysis if we mix these ACEIs.

Previous studies suggest that the ARB therapy in patients with a normal kidney function or chronic kidney disease without dialysis might protect heart and kidney from disorders. A randomized controlled trial study showed that ARBs didn’t significantly lower the risks of major cardiovascular events or death among patients with hypertension on chronic HD [22]. A meta-analysis study concluded that ARB wasn’t associated with a statistically significant reduction in the risk of fatal and nonfatal CV events [23]. An open-label randomized controlled trial study proved ARBs to be effective in reducing nonfatal CVD events in patients undergoing a long-term HD [22]. One study revealed that blockade of rennin-angiotensin system might be effective in heart failure patients on long-term Hemodialysis [24]. This is the first ARB study focused on attenuating the risk of MACE in ESRD patients. In Fig 2, we find the association of ARB use with decreased risk of MACE was significant in the ARB (++) group (more than 90 cumulative prescription days), as compared to the ARB (+) (under 90 cumulative prescription days) or ARB (-) group (no ARB treatment). Furthermore, subdividing the groups by cumulative prescription days shows that longer total treatment duration is crucial for decreasing risk for MACE, with more than 365 treatment days associated with significantly decreased risk for MACE.

The use of ARB in patients on maintenance dialysis focuses on blood pressure control. After using ARB, the number of dialysis patients that need to do invasive cardiovascular examination was decreased significantly. However for some reason, such as hyperkalemia, 537 patients with hypertension in this study do not be prescribed ARBs for blood pressure control in Taiwan (Table 1). We should encourage nephrologists to prescribe ARBs as the first-line medications for blood pressure control.

In Table 2, we found that hypertension alone is not an independent factor to the development of MACE in maintenance dialysis patients after adjusted for gender, age, and comorbidities (HR = 1.12, 95% CI = 0.94–1.33, P = 0.196). Besides the hypertension, diabetes mellitus and hyperlipidemia still increase the risk of MACE in these patients (HR = 1.40, 95% CI = 1.21–1.63, P < 0.001; and HR = 1.27, 95% CI = 1.09–1.49, P < 0.05, respectively).

Table 3 show the MACE protection by ARBs correlates with the cumulative prescription days of ARBs. In short-term use group ARB (+) there is no difference of MACE occurrences compared with non-user group ARB (-) (HR = 1.00, 95% CI = 0.81–1.23). In long-term use group ARB (++), a significant decrease of MACE was noted when cumulative prescription days of ARB > 365 days.

Table 4 show the difference comorbidities influence the effect of ARBs to reduce the risk of MACE. For patients with hypertension only, longer cumulative prescription days (> 1095 days) are needed to reduce the risk (HR = 0.26, 95% CI = 0.08–0.81, P < 0.05). For patients with hypertension and adding diabetes mellitus or hyperlipidemia, cumulative prescription days over 365 days can reach the target to reduce the risk of MACE. However, for patients with hypertension and hyperlipidemia, the benefits vanishes when cumulative prescription days > 1095 days. For patient with hypertension, diabetes mellitus, and hyperlipidemia, benefits of MACE reduction occur earlier after prescription of ARBs over 90 days and these benefits continue.

In Table 5, we find that the long-term use of ARBs can reduce the risks of AMI, acute stroke, PTA for PAD patients, but there are no obvious benefits on patients of CAD for elective PTCA procedures.

The mechanisms by which ARBs protect hypertensive dialysis patients from MACE are still not completely clear. Direct blood pressure control is surely a crucial factor [25]. However, other studies have reported several effects independent of blood pressure control, such as modulation of endothelial function or arteriosclerotic plaques [26–28]. Some researchers have found that AT_1_-receptor activation could induce superoxide radical release via the NAPDH oxidase pathway, increasing oxidative stress, and thus leading to endothelial dysfunction [29, 30]. Therefore, ARBs may play a role in protecting the endothelium by blocking the AT_1_-receptor, thus preventing the formation of endothelial atherosclerotic plaque in the arteries. ARBs could also modulate or decrease atherosclerotic plaque in arteries [31, 32]. Protection of the endothelium or modulation of atherosclerotic plaque might contribute to the prevention of AMI and acute stroke. However, this study showed that ARB therapy might not decrease the demand of PTCA for CAD patients. Further studies are needed to clarify why this group of patients seems to not benefit from ARB therapy.

This study is subject to certain limitations. The claim databank provides only the number of ARB prescription days, but contains no data for blood pressure or other risk factors for MACE including obesity, smoking, alcohol consumption, family history, lifestyle, and diet or genetic markers. Although this is an important limitation in our study, we have done comorbidity-relation analysis for MACE as shown in Table 4. The blood pressure records and the comorbidities, such as hypertension, hyperlipidemia and diabetes mellitus, are metabolic risk factors which are related to the factors of obesity, smoking, alcohol consumption, family history, lifestyle, and diet or even genetic markers. Since this study has adjusted these factors, considerable part of these factors should have been adjusted. In addition, we have no way of knowing if patients actually took their prescribed medication regularly. Death certificate data or autopsy reports are not included in the NHIRD dataset, either. Therefore, MACE-related mortality could not be analyzed.

## Conclusions

For patients on maintenance dialysis, ARB therapy for hypertension could attenuate the risk of MACE, especially for acute myocardial infarctions, acute strokes, and PAD requiring PTA. More than 365 cumulative ARB prescription days are needed to lower the risk of MACE, except for patients with comorbidities of both diabetes mellitus and hyperlipidemia who benefitted from > 90 cumulative prescription days. Further studies are needed to clarify the ARB mechanisms for risk attenuation for MACE and the effects on MACE-related mortality in maintenance dialysis patients.
